# Improved production of poly(lactic acid)-like polyester based on metabolite analysis to address the rate-limiting step

**DOI:** 10.1186/s13568-014-0083-2

**Published:** 2014-11-18

**Authors:** Ken’ichiro Matsumoto, Kota Tobitani, Shunsuke Aoki, Yuyang Song, Toshihiko Ooi, Seiichi Taguchi

**Affiliations:** Division of Biotechnology and Macromolecular Chemistry, Graduate School of Engineering, Hokkaido University, N13-W8, Kita-ku, Sapporo, 060-8628 Japan; PRESTO-JST, K's Gobancho, Building 7, Gobancho Chiyoda-ku, Tokyo, 102-0076 Japan; College of Enology, Northwest A&F University, 22 Xinong Road, Yangling, Shaanxi 712100 China; CREST, JST, 4-1-8 Honcho, Kawaguchi, 332-0012 Saitama, Japan

**Keywords:** Biobased plastic, P(LA-co-3HB), Polyhydroxybutyrate, Metabolome analysis

## Abstract

The biosynthesis of poly(lactic acid) (PLA)-like polymers, composed of >99 mol% lactate and a trace amount of 3-hydroxybutyrate, in engineered *Corynebacterium glutamicum* consists of two steps; the generation of the monomer substrate lactyl-coenzyme A (CoA) and the polyhydroxyalkanoate (PHA) synthase-catalyzed polymerization of lactyl-CoA. In order to increase polymer productivity, we explored the rate-limiting step in PLA-like polymer synthesis based on quantitative metabolite analysis using liquid chromatography mass spectroscopy (LC-MS). A significant pool of lactyl-CoA was found during polymer synthesis. This result suggested that the rate-limitation occurred at the polymerization step. Accordingly, the expression level of PHA synthase was increased by means of codon-optimization of the corresponding gene that consequently led to an increase in polymer content by 4.4-fold compared to the control. Notably, the codon-optimization did not significantly affect the concentration of lactyl-CoA, suggesting that the polymerization reaction was still the rate-limiting step upon the overexpression of PHA synthase. Another important finding was that the generation of lactyl-CoA was concomitant with a decrease in the acetyl-CoA level, indicating that acetyl-CoA served as a CoA donor for lactyl-CoA synthesis. These results show that obtaining information on the metabolite concentrations is highly useful for improving PLA-like polymer production. This strategy should be applicable to a wide range of PHA-producing systems.

**Electronic supplementary material:**

The online version of this article (doi:10.1186/s13568-014-0083-2) contains supplementary material, which is available to authorized users.

## Introduction

Bacterial polyesters polyhydroxyalkanoates (PHAs) are synthesized via the supply of monomer hydroxyacyl-CoA molecules and polymerization of the monomers catalyzed by PHA synthases (Rehm [2003]; Matsumoto and Taguchi [2013b]; Lu et al. [2009]). The rate-limiting step in PHA synthesis may be either the monomer supply or polymerization, which can vary depending on the combination of the relevant enzymes, production hosts and carbon source. To date, the rate-limiting step has been estimated by modulating the activity of each step to see its effect on polymer productivity (Jung et al. [2000]; Kichise et al. [1999]; Taguchi et al. [2001]). This indirect approach, however, was unable to provide quantitative information on the metabolic pathways. The aim of this study was to determine the intracellular concentration of the metabolic intermediates in the PHA biosynthetic pathways in order to explore the rate-limiting step.

**Figure 1:**
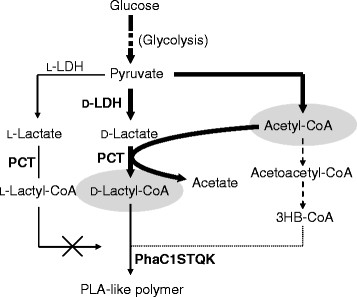
**Metabolic pathway for PLA-like polymer production in engineered** ***C. glutamicum*** **.** PLA-like polymers contained <1 mol% 3HB units. L-LDH, L-lactate dehydrogenase; D-LDH, D-lactate dehydrogenase; PCT, propionyl-CoA transferase; PhaC1STQK, lactate-polymerizing PHA synthase. The bold letters indicate exogenous enzymes. The dashed lines indicate the putative pathways. The dotted line indicates a very weak pathway. The gray ovals indicate significantly pooled metabolites.

In this study, we chose the PLA-like polymer-producing *Corynebacterium glutamicum* as the target (Song et al. [2012]). This microorganism, which is known as an industrial amino acid producer with GRAS (Generally Regarded As Safe) status, has been engineered to express three exogenous genes encoding D-lactate dehydrogenase, propionyl-CoA transferase (PCT) and LA-polymerizing PHA synthase (PhaC1_Ps_STQK) (Taguchi and Doi [2004]; Taguchi et al. [2008]) (see pathway in Figure 1). This unique bacterial system was shown to be capable of producing PLA-like polymer directly from glucose via one-pot fermentation. It should be noted that the PLA-like polymer consists of >99 mol% LA and a trace amount of 3-hydroxybutyrate (3HB) (Song et al. [2012]), and thus is here referred to as PLA'. The challenge of the system was that had to be overcome was the low productivity of the polymer (0.03 g/L) compared to typical bacterial PHAs.

To meet this challenge, we attempted to identify the rate-limiting step in PLA' synthesis by means of quantitative metabolite analysis using liquid chromatography mass spectroscopy (LC-MS) (Zhou et al. [2012]). This method was reported to be suitable for measuring the derivatives of CoA having a relatively high molecular weight and polarity, while gas chromatography-MS has been used for detecting relatively low-polarity and small molecules (Poblete-Castro et al. [2012]). In this study, the concentrations of the important intermediates for PLA' production were determined, i.e. lactyl-CoA, acetyl-CoA, acetoacetyl-CoA and 3HB-CoA (Figure 1). To the best of our knowledge this was the first successful monitoring of the intracellular CoA-derivatives during PLA' production, which was realized by a rational metabolic engineering approach.

## Materials and methods

### Plasmid construction

The four oligonucleotides 5'-gtcaccggatcccggttaactctag-3', 5'-actcgagcctgcaggagatcttcgatatca-3', 5'-ctagtgatatcgaagatctcctgc-3' and 5'-aggctcgagtctagagttaaccgggatccg-3' were inserted into the *Xba* I/*Bst* EII sites of pPSPTG1 (Song et al. [2012]) so as to create *Bam* HI, *Hpa* I, *Xba* I, *Xho* I, *Sbf* I, *Bgl* II and *Eco* RV sites (pPSDCP). Fragments of the *ldhA* gene from *Escherichia coli* (gene ID: 12930508), the *pct* gene from *Megasphaera elsdenii* and the *phaC1STQK* gene (Taguchi et al. [2008]) were inserted into pPSDCP at *Bam* HI/*Hpa* I, *Bgl* II/*Eco* RV and *Xba* I/*Bgl* II sites, respectively, to yield pPS*ldhAC1STQKpct*. The codon-optimized *phaC1STQK* gene [*ephaC1STQK*, accession No. AB983346 (DDBJ)] was chemically synthesized (Eurofins Genomics) for the expression in *C. glutamicum* and its *Xba* I/*Sbf* I fragment was inserted in a similar manner so as to yield pPS*ldhAeC1STQKpct* (Additional file 1: Table S1).

### Strain, culture conditions and polymer analysis

*C. glutamicum* ATCC13803 was transformed by electroporation, as described previously (Liebl et al. [1989]). For polymer production, the engineered strains were grown in 2 ml nutrient-rich CM2G medium (Kikuchi et al. [2003]) at 30°C for 24 h with reciprocal shaking at 180 strokes/min. Two hundred microliters of the preculture were then transferred into 2 mL minimal MMTG medium (Kikuchi et al. [2003]) containing 60 g/L glucose and 0.45 mg/L of biotin, and further cultivated for 72 h at 30°C. When needed, kanamycin (50 μg/mL) was added to the medium. After cultivation, cells were lyophilized for polymer extraction. The polymer content was determined using gas chromatography as described previously (Takase et al. [2004]). Based on this analytical method, 3HB units in the polymer were below the detection limit.

### LC-MS analysis

The cell extract was prepared using a method modified from a previous report (Kiefer et al. [2011]). The cells were cultivated on 2 mL MMTG medium as described above, then harvested at 18 h. The cells were resuspended in 200 μL of chilled water, then combined with 1 mL chilled acetonitrile containing 0.1 M formic acid and treated with sonication for 5 sec × 5 times. The supernatant was transferred to a new microtube and evaporated *in vacuo* at 4°C. The sample was dissolved in 200 μL of chilled water. LC-MS analysis was performed using an LCMS-8030 (Shimadzu) equipped with a Mastro C18 column (150 mm), electrospray ionization (ESI) and triple quadrupole mass spectroscopy. Carrier A: 5 mM ammonium acetate (pH 5.6) containing 5 mM dimethylbutylamine (Gao et al. [2007]) and carrier B: methanol were used with a flow rate of 0.2 mL/min in gradient mode, as follows: 0 min, 10% B; 3 min, 10% B; 15 min, 95% B; 18 min, 95% B; 23 min, 10% B. The ESI voltage was 3.5 kV in the negative mode. Nitrogen was used as a nebulizer (3.0 mL/min) and drying gas (15.0 mL/min). [M-H]^-^ ions from acetyl-CoA (m/z = 808, retention time: 9.1 min), acetoacetyl-CoA (m/z = 850, rt: 8.9 min), 3HB-CoA (m/z = 852, rt: 9.1 min) and lactyl-CoA (m/z = 838, rt: 8.7 min) were monitored using the selected ion monitoring mode. Acetyl-CoA, acetoacetyl-CoA and 3HB-CoA, used as standards, were purchased from Sigma Aldrich. Lactyl-CoA was synthesized via CoA-transferring reaction by PCT, as follows. The reaction mixture containing 100 mM Tris-HCl (pH 7.4), 0.4 mM acetyl-CoA, 12.5 mM sodium lactate and 0.1 mg/mL purified His-tagged PCT (Additional file 1) was incubated at 30°C for 30 min. Then lactyl-CoA was purified using a preparative HPLC equipped with a C18 reverse phase column.

### SDS-PAGE analysis

The cells cultivated under the polymer producing conditions were harvested at 18 h. The cells were resuspended in 25 mM Tris-HCl (pH 7.5) buffer and treated with sonication. The whole cell extracts were subjected to SDS-PAGE.

## Results

### The use of a single plasmid system increased transformation efficiency

In a previous study, a dual plasmid system using pPS and pVC vectors was used for PLA' production (Song et al. [2012]). To reduce the use of antibiotics, the expression vector was reconstructed so that the recombinant form was maintained only in the presence of kanamycin. In addition, the single plasmid system improved the transformation efficiency compared to the dual plasmid system (from 3 × 10^2^ to 2 × 10^4^ colonies/μg DNA). Therefore, the single plasmid system was used for further study.

### Determination of the CoA intermediates in the engineered *C. glutamicum*

**Figure 2:**
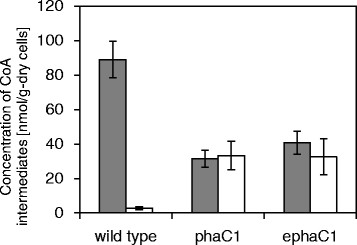
**Concentrations of the CoA derivatives in** ***C. glutamicum*** **during PLA-like polymer production.** The cells were harvested at 18 h. The data is the average of six independent samples along with the standard deviation. Gray: acetyl-CoA, white: lactyl-CoA.

The wild-type *C. glutamicum* and the recombinant cells harboring pPS*ldhAC1STQKpct* were cultivated under the polymer-producing conditions. The cells were harvested in the logarithmic growth phase (18 h) and subjected to metabolite analysis. The concentrations of aectyl-CoA, lactyl-CoA, acetoacetyl-CoA and 3HB-CoA in the cells were determined. The key points for the successful measurement of the CoA derivatives were shown to be a rapid extraction of the cells along with the an appropriate ion pair reagent. For the wild-type cells, the acetyl-CoA concentration was 89 nmol/g-dry cells, and unexpectedly, a small amount of lactyl-CoA was also observed (Figure 2). In contrast, the recombinant cells exhibited an elevated concentration of lactyl-CoA, which was concomitant with a significant reduction in the concentration of acetyl-CoA. This result suggests that PCT promoted lactyl-CoA synthesis, and more importantly, acetyl-CoA is able to serve as a CoA donor for the generation of lactyl-CoA (Figure 1). The concentrations of acetoacetyl-CoA and 3HB-CoA were below the detection limit for all of the conditions tested (data not shown). From these observations, the interconversion of lactate + acetyl-CoA ↔ lactyl-CoA + acetate presumably achieved an equilibrium state, namely, the polymerization of lactyl-CoA would be a retarded step.

### Enhanced expression of PHA synthase elevated PLA-like polymer production

**Figure 3:**
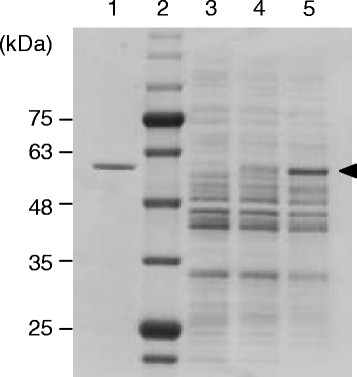
**SDS-PAGE analysis of** ***C. glutamicum*** **.** Whole cell extracts were applied. The arrow indicates the size of PhaC1STQK. 1: Purified His-tag fusion of PhaC1STQK (0.072 μg), 2: size marker, 3: wild type (5.4 μg), 4: recombinant form harboring the parent PHA synthase gene *phaC1STQK* (5.0 μg), 5 : recombinant form harboring the codon-optimized *ephaC1STQK* gene (4.0 μg).

In order to evaluate the aforementioned hypothesis and to overcome the existing limitation, we attempted to improve the expression level of PHA synthase. For this purpose, the codon-optimized PHA synthase gene *ephaC1*_Ps_STQK, which was supposed to be efficiently translated in *C. glutamicum*, was synthesized. First, the effect of the codon-optimization on the expression of the enzyme was evaluated. As shown in Figure 3, the cells harboring the codon-optimized gene had an increase in the expression level of PHA synthase.

Next, the PLA' production was investigated for the cells harboring parent and codon-optimized PHA synthase genes. As expected, the cells expressing a higher level of PHA synthase accumulated more PLA' (4.4-fold) than the control (Table 1). Thus, the metabolic engineering, which was designed based on the metabolite analysis, did successfully improve the polymer production. In addition, this result supported the validity of the method for the determination of the metabolite concentrations.

**Table 1:** **PLA-like polymer production in engineered** ***C. glutamicum*** ^**a**^

Relevant genes	Cell dry weight (g/L)	Polymer content (wt%)
None (wild-type control)	11.4 ± 1.7	ND^b^
*phaC1STQK, pct, ldhA*	10.7 ± 2.0	0.27 ± 0.01
*ephaC1STQK, pct, ldhA*	9.3 ± 0.5	1.19 ± 0.01

^a^Cells were grown on 2 mL MMTG medium containing glucose for 72 h at 30°C. The data is the average of triplicate samples along with the standard deviation. ^b^ND: not detected.

### Metabolite analysis of the modified cells indicated the rate-limiting step remained

The metabolite concentrations in the cells harboring the codon-optimized gene were measured. Despite the increase in the polymer content (Table 1), there was no significant difference between the metabolite levels in the two types of recombinant cells (Figure 2). This result indicated that the rate-limiting at polymerization step remained even with the reinforced expression of PHA synthase.

## Discussion

In this study, metabolite analysis was shown to provide quantitative information that was very useful for addressing the rate-limiting step in the PLA' production. In the previous studies, the rate-limiting step in PHA production has been only qualitatively explored based on the polymer production *in vivo*. For examples, in the case of P(3HB) production in *C. glutamicum*, the expression of the highly active acetoacetyl-CoA reductase (PhaB) mutant from *Ralstonia eutropha* improved P(3HB) production (Matsumoto et al. [2013]), suggesting that monomer supply is a rate-limiting step in P(3HB) synthesis. A similar result was obtained in transgenic P(3HB)-producing tobacco (Matsumoto et al. [2011]). Here it should be noted that in these studies it was impossible to determine whether the improved the PhaB activity was sufficient or not unless the PhaB activity was further increased. In other words, P(3HB) production should reach a plateau if the PhaB activity was sufficient under the original conditions. In contrast, based on the metabolite analysis, the rate-limitation of PLA' synthesis at polymerization step was shown to remain after PHA synthase was overexpressed. Therefore, it was expected that an additional enhancement in polymerizing activity would be needed to increase PLA' production.

It was a chance discovery that the engineered *C. glutamicum* expressing PhaC1_Ps_STQK synthesized PLA'. On the other hand, it has been reportedly demonstrated that *E. coli* engineered to express the same set of enzymes did not produce PLA' (Nduko et al. [2013]; Shozui et al. [2011]; Yamada et al. [2009]; Matsumoto and Taguchi [2013a]). Thus, the mechanism for PLA' synthesis in *C. glutamicum* has been an important issue. A clue for answering this question might be the small amount of 3HB units (<1 mol%) incorporated into the polymer (Song et al. [2012]). The presence of 3HB units in the polymer suggests that this organism is likely to possess intrinsic 3HB-CoA (Figure 1). The result of the present study, however, demonstrated that the concentration of 3HB-CoA was below the detection limit. Thus, 3HB-CoA may be synthesized via an unidentified, very weak route and/or rapidly metabolized. In comparison, in *R. eutropha*, which is an efficient P(3HB) producer, a much higher concentration of 3HB-CoA (0.1-1 nmol/g-dry cells) has been reportedly observed (Fukui et al. [2014]). The very low 3HB-CoA level in *C. glutamicum* might account for the capacity of this organism to produce the copolymer with an extremely high LA fraction. Further investigation of LA-based polymer-producing *E. coli* is needed to clarify this issue.

The wild-type *C. glutamicum* unexpectedly synthesized lactyl-CoA or another compound having the same m/z and retention time. The metabolite level was too low to be detected using the MS/MS mode. Thus, currently the molecule is not confidently identified. However, if the existence of lactyl-CoA is postulated, this molecule should be L-lactyl-CoA, which cannot be incorporated into the polymer due to the strict stereospecificity of PHA synthase (Tajima et al. [2009]), because the wild-type *C. glutamicum* possesses no D-LDH gene (Kalinowski et al. [2003]). To date, the presence of lactyl-CoA in *C. glutamicum* has not been reported and its physiological role is unknown. Thus it may be an interesting research target.

Although PLA' production was successfully increased by the overexpression of PHA synthase (Table 1), the polymer content was lower than previously reported results (up to 1.4 wt%) obtained using100 mL-scale flask cultures (Song et al. [2012]). This difference was probably due to the aeration efficiencies in the test tubes and flasks. Because the lactic acid production in *C. glutamicum* is influenced by the oxygen supply (Inui et al. [2004]), the aeration rate would be expected to have an impact on PLA' production. More detailed analysis and fine-tuning of the aeration using a jar-fermentor is needed to optimize the culture conditions.

In summary, the levels of CoA derivatives in engineered *C. glutamicum* during PLA' production were determined, which allowed us to identify a rate-limitation at the polymerization step. In fact, overexpression of PHA synthase successfully increased the polymer production. In addition, acetyl-CoA probably served as a CoA donor for supplying lactyl-CoA in *C. glutamicum*.

## Additional file

## Electronic supplementary material

Additional file 1: Protocol for purification of PCT. (DOCX 45 KB)

Below are the links to the authors’ original submitted files for images.

Authors’ original file for figure 1

Authors’ original file for figure 2

Authors’ original file for figure 3
