# Supplementary material for: Improved production of poly(lactic acid)-like polyester based on metabolite analysis to address the rate-limiting step
Source: AMB Express. 2014 Nov 18;4:83. doi: 10.1186/s13568-014-0083-2 (PMC4884051; doi:10.1186/s13568-014-0083-2)
Supplement: Supplementary file 1 — Additional file 1: Protocol for purification of PCT. (DOCX 45 KB) [file 13568_2014_83_MOESM1_ESM.docx]

**Supplemental materials**

**"Improved production of poly(lactic acid)-like polyester based on metabolite analysis to address the rate-limiting step"**

**Ken'ichiro Matsumoto, Kota Tobitani, Shunsuke Aoki, Yuyang Song, Toshihiko Ooi and Seiichi Taguchi**

**Table S1. The list of plasmids used in this study**

| Plasmid name | Relevant genes | Reference |
| --- | --- | --- |
| pPSPTG1 | Transglutaminase gene with *cspB* promoter | Kikuchi et al. 2013 |
| pPSDCP | pPSPTG1 derivative; multi-cloning sites | This study |
| pPS*ldhC1STQKpct* | pPSDCP derivative; *ldhA* gene from *E. coli*, *phaC1*_Ps_*STQK* gene from *Pseudomonas* sp. 61-3, and *pct* gene from *M. elsdenii* | This study |
| pPS*ldheC1STQKpct* | pPSDCP derivative; *ldhA* gene from *E. coli*, codon-optimized *ephaC1*_Ps_*STQK* gene from *Pseudomonas* sp. 61-3, and *pct* gene from *M. elsdenii* | This study |


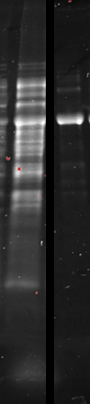


**1 2**

**Figure S1. Protocol for purification of PCT.** Recombinant *E. coli* JM109 harboring pQE30 (Qiagen), which includes His-tagged PCT at N-terminal, was grown on 1.5 mL LB medium containing 100 µg/l ampicillin at 37 °C for 15 h (preculture). The cells (1 mL) were transferred to 100 mL of the same medium and grown at 30 °C for 2 h. Then, the flasks were transferred to 25 °C incubator and 1 mM IPTG (final concentration) was added to the flask. The cells were further cultivated at 25 °C for 24 h. The cells were harvested, and disrupted by sonication in lysis buffer [50 mM sodium phosphate (pH 8.0) containing 10 mM imidazole and 300 mM sodiuim chloride]. The supernatant was combined with 500 µL of Ni-NTA His-Bind Resins (Novagen), and gently rotated at 4 °C for 1 h. The resin was applied to an empty column, and washed with 1 mL of lysis buffer, and subsequently, washed with 5 mL of lysis buffer but containing 20 mM imidazole. The bound protein was eluted with 4 mL of lysis buffer containing 250 mM midazole, and immediately desalted using a PD-10 column (GE healthcare) with 50 mM sodium phosphate buffer (pH 7.0) containing 5% glycerol. The purified protein solution was frozen using liquid nitrogen and stored at -80 °C. Lane 1: crude extract. Lane 2: purified PCT.
