# Supplementary figures and images for: Improved production of poly(lactic acid)-like polyester based on metabolite analysis to address the rate-limiting step
Source: AMB Express. 2014 Nov 18;4:83. doi: 10.1186/s13568-014-0083-2 (PMC4884051; doi:10.1186/s13568-014-0083-2)

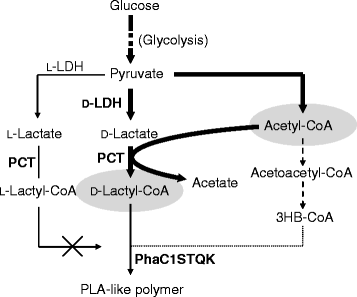

Supplement: Supplementary file 2 — Authors’ original file for figure 1 [file 13568_2014_83_MOESM2_ESM.gif]

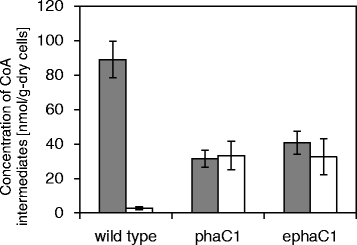

Supplement: Supplementary file 3 — Authors’ original file for figure 2 [file 13568_2014_83_MOESM3_ESM.gif]

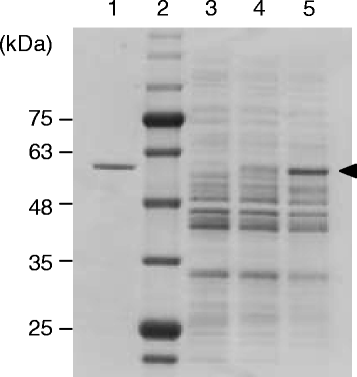

Supplement: Supplementary file 4 — Authors’ original file for figure 3 [file 13568_2014_83_MOESM4_ESM.gif]
